# Eliciting the Level of Health Inequality Aversion in England

**DOI:** 10.1002/hec.3430

**Published:** 2016-09-20

**Authors:** Matthew Robson, Miqdad Asaria, Richard Cookson, Aki Tsuchiya, Shehzad Ali

**Affiliations:** ^1^ Department of Economics and Related Studies University of York York UK; ^2^ Centre for Health Economics University of York York UK; ^3^ Department of Economics, and School of Health and Related Research University of Sheffield Sheffield UK; ^4^ Department of Health Sciences University of York UK

**Keywords:** health inequality, inequality aversion, social preferences, survey, welfare function

## Abstract

Health inequality aversion parameters can be used to represent alternative value judgements about policy concern for reducing health inequality versus improving total health. In this study, we use data from an online survey of the general public in England (*n* = 244) to elicit health inequality aversion parameters for both Atkinson and Kolm social welfare functions. We find median inequality aversion parameters of 10.95 for Atkinson and 0.15 for Kolm. These values suggest substantial concern for health inequality among the English general public which, at current levels of quality adjusted life expectancy, implies weighting health gains to the poorest fifth of people in society six to seven times as highly as health gains to the richest fifth. © 2016 The Authors. *Health Economics* published by John Wiley & Sons, Ltd.

## 1 Introduction

Improving total population health and reducing health inequality are two important objectives of health policy. When these two objectives conflict, Health‐Related Social Welfare Functions (HRSWFs) can be used to articulate the trade‐off between them. Economists have explored the properties of several SWFs, which aim to capture these trade‐offs in the form of a single inequality aversion parameter. The Atkinson Index (Atkinson, 1970) and Kolm Index (Kolm, 1976) are two such forms, concerned with relative and absolute concepts of inequality widely used in the income inequalities literature. A range of two person HRSWFs have also been proposed (Wagstaff, 1991; Abásolo and Tsuchiya, 2004), and there have been various attempts to elicit health inequality aversion parameters for some of these functions from interview data of members of the public in England (Dolan and Tsuchiya, 2011; Edlin *et al*., 2012) and Spain (Abásolo and Tsuchiya, 2013).

Building on the questionnaire instrument employed in these existing studies, we have previously conducted methodological work to develop and validate a video animation that vividly presents different viewpoints and their implications on distribution of health, designed to encourage respondents to think carefully about their responses (Cookson *et al*., 2015). The contribution of this paper is to elicit inequality aversion parameters for the Atkinson and Kolm SWFs by using online survey data from the general population in England that incorporates this video animation. These parameter values can help to inform health policy makers in England who wish to explicitly incorporate social value judgements concerning health inequalities into decisions regarding the allocation of healthcare resources.

## 2 Methods

### 2.1 Survey

Details of the survey methods and sample selection are reported in Appendix A in the Supporting Information. Respondents were presented with information highlighting inequalities in expected years of life in full health at birth between the richest and poorest fifths of people in England. Respondents made a series of seven pairwise choices between two programmes, which would increase expected years in full health. In each choice, Programme A favoured the richest fifth and Programme B the poorest fifth. In the first choice, Programme A provided an increase of seven years to the rich and three years to the poor and Programme B provided an increase of three years to the rich and eight years to the poor. In each successive choice, the years gained by the poor group in Programme B were gradually reduced, while everything else was fixed. In each choice, the respondents were asked to decide whether the government should choose Programme A, Programme B or whether the two programmes were ‘equally good’.

### 2.2 Categorisation

To elicit the inequality aversion parameters, we developed a response classification system, which is shown in Appendix B. The point at which the respondent ‘switches’, or becomes indifferent, between the programmes was used to categorise respondents and derive the level of inequality aversion. Those categorised as ‘Pro‐Rich’ prefer health gains to the better‐off, ‘Health Maximisers’ are concerned only with increasing total health, ‘Weighted Prioritarians’ give greater weight to the health of the worse‐off, ‘Maximin’ respondents are concerned only with improving the health of the worst‐off and ‘Egalitarians’ value reducing health inequality so much that they are willing to sacrifice potential health benefits to the worst‐off.

**Figure 1:**
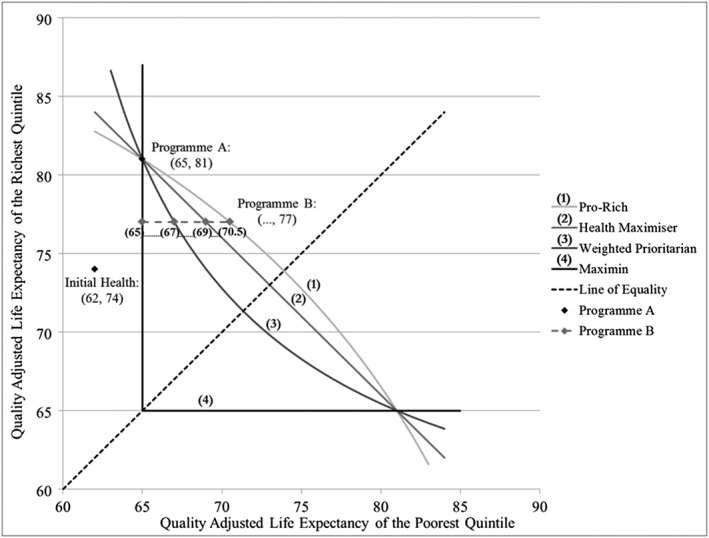
Iso‐welfare curves representing response categories

Figure 1 illustrates a range of iso‐welfare curves for the Atkinson Index, plotted at different levels of inequality aversion corresponding to four selected responses. The horizontal and vertical axes represent the quality‐adjusted life expectancy of the poorest and the richest fifths, respectively. Point (62,74) is the initial distribution of health, while point (65,81) represents Programme A, which remains fixed through the seven choices. The dashed line from (65,77) to (70.5,77) gives the range of values from Programme B.

### 2.3 Social welfare functions and equally distributed equivalents

The equally distributed equivalent (EDE) level of health builds on the concept of EDE income, defined as the mean level ‘which if equally distributed would give the same level of social welfare as the present distribution’ (Atkinson, 1970). It provides an index of social welfare standardised to the mean level of health, which enables the comparison of different health distributions. The equations for the EDEs used in this paper are as follows:

(1) $$EDE_{\text{Atkinson}}=\bar{H}.{\left[\sum_{i}{\left(\frac{H_{i}}{\bar{H}}\right)}^{1-\varepsilon }\;f\left(x_{i}\right)\right]}^{1/\left(1-\varepsilon \right)}$$

(2) $$EDE_{\text{Kolm}}=\bar{H}-\left[\left(\frac{1}{\alpha }\right)\text{log}\sum e^{\alpha .\left(\bar{H}-H_{i}\right)}f\left(x_{i}\right)\right]$$

In these equations, *ε* and *α* are the inequality aversion parameters for the Atkinson and Kolm HRSWFs respectively, where both are unbounded. The greater the value of *ε* and *α*, the greater the aversion to inequality. *H*_*i*_ is the level of health (quality‐adjusted life expectancy) for subgroup *i*, $\bar{H}$ is the mean level of health for the entire population and *f*(*x*_*i*_) is the proportion of the population in subgroup *i*. Unlike most previous studies, our data allow the elicitation of negative parameters for individual respondents representing ‘inequality seeking’ judgements and positive parameters representing ‘inequality averse’ ethical judgements.

### 2.4 Parameter elicitation

The point where a given respondent switches from one programme to the other, or selects equally good, is interpreted to reflect the point at which the respondent is indifferent between the two programmes. The implied inequality aversion parameter for each respondent can then be established by numerically solving the EDE equations.

### 2.5 Establishing a population average

In order to represent an inequality aversion parameter for the population, we use the median response rather than the mean. This is because the inequality aversion parameter approaches infinity for Maximin responses (Dolan and Tsuchiya, 2011). To make the analysis sample as representative as possible, population weights for England for age, gender, income and education were derived from Understanding Society Wave 4 (Essex., 2015) and used to weight sample responses to construct a response set representative of the general population. Further details of this weighting can be found in Appendix C. To allow for uncertainty, we estimate 95% confidence intervals for each parameter by using a non‐parametric bootstrap of the survey data, resampling from the population weighted raw data 2000 times.

### 2.6 Sensitivity analyses

Our classification scheme focused on ‘logical’ response patterns, in which only one ‘switch’ is observed or one programme is selected throughout, and the results reported are for those responses that were consistent with this classification. We also conducted sensitivity analyses by using more permissive inclusion criteria, as explained in Appendix D.

## 3 Results

**Figure 2:**
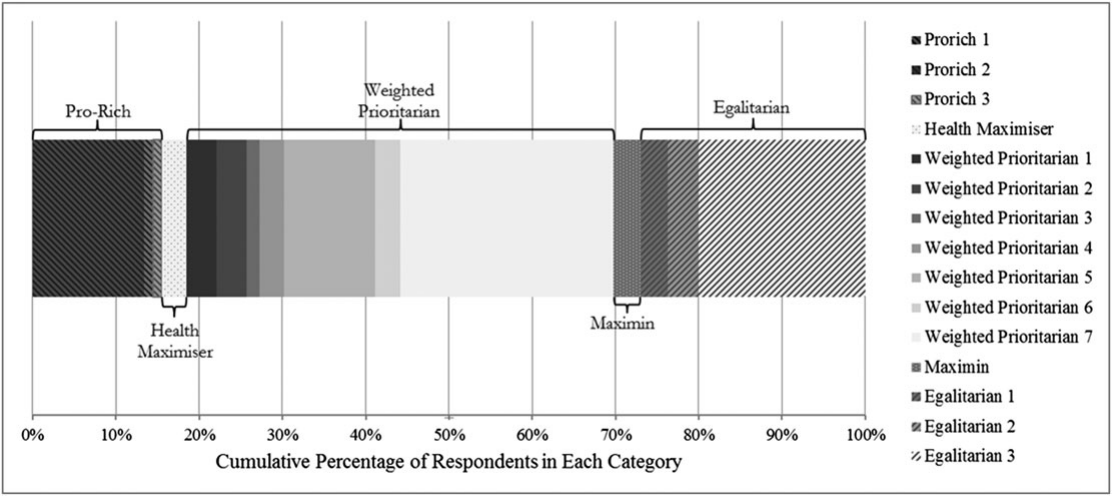
Weighted distribution of categorical responses (*n* = 244)

An online survey generated usable data from 244 respondents whose data were used in the base case analysis (see Appendix C for the characteristics of the analysis sample and Appendix D for a breakdown of those excluded for providing invalid data). Figure 2 illustrates the distribution of responses for each category, after sample re‐weighting. Just over half, 50.98%, of the responses were Weighted Prioritarian, but there were substantial responses in the tails of the distribution with 15.58% being Pro‐Rich and 26.98% being Egalitarian. The vast majority of respondents, 81.51%, were willing to trade‐off some total health in order to reduce health inequality and only 2.91% were strict Health Maximisers. The parameters for each categorical response, for each SWF, can be found in Appendix E.

The elicited inequality aversion parameters, and the EDE level of health they imply, are given in Table 1. The results imply that at initial levels of quality‐adjusted life expectancy, incremental health gains to the poorest fifth of people in society should be weighted between 6 and 7 times as highly as incremental health gains to the richest fifth. Sample weighted bootstrapping revealed that the 95% confidence intervals did not exceed the category in which the median response was located. Furthermore, the median response did not change with the population weighting.

**Table 1:** Base case inequality aversion parameters with 95% confidence intervals

SWF	Median^a^	Implied weight^b^
	(95% CI)	(95% CI)
Atkinson (*ε*)	10.95	6.95
	(10.95–10.95)	(6.95–6.95)
Kolm (*α*)	0.15	6.20
	(0.15–0.15)	(6.20–6.20)

a Median preference identified through bootstrapping; population weights used.

b Implied weight of marginal health gain to poorest fifth of the population compared with the marginal health gain to the richest fifth of the population at initial health.

The median inequality aversion parameters were robust to sensitivity analyses using larger samples (see Appendix D for details).

## 4 Discussion

### 4.1 Comparison to previous literature

There have been a number of previous empirical studies of health inequality aversion using the same basic questionnaire instrument in England (Williams *et al*., 2005; Dolan and Tsuchiya, 2007; Dolan and Tsuchiya, 2009; Dolan and Tsuchiya, 2011) and Spain (Abásolo and Tsuchiya, 2004; Abásolo and Tsuchiya, 2008; Abásolo and Tsuchiya, 2013). All of these studies have found that the majority of the population is willing to sacrifice a substantial amount of total health in order to reduce health inequality, as have most studies using different instruments (Edlin *et al*., 2012; Attema *et al*., 2015). In most cases, it is not possible to extract comparable inequality aversion parameter central estimates, because parameters were not reported or are not comparable. However, Dolan and Tsuchiya (2011) report an equivalent Atkinson *ε* value of 28.9, which is considerably larger than our value of 10.95. There are multiple factors that could explain this difference. One may be that their study did not use a ‘slow thinking’ intervention (such as our e‐learning video animation; Cookson *et al*., 2015), which may reduce the proportion of respondents expressing extreme inequality aversion (Appendix F); another may be that their study used face‐to‐face rather than online administration, which is more likely to be associated with social desirability bias leading to more extreme egalitarian responses; a third may be that their study used half‐year response categories, which allow more extreme inequality aversion to be expressed. Two of the studies in Spain, Abásolo and Tsuchiya (2004) and Abásolo and Tsuchiya (2013), found such extreme inequality aversion that the Atkinson *ε* value was not identifiable as the median response violated monotonicity—i.e. Programme B was selected in all choice pairs.

### 4.2 Application to distributional cost‐effectiveness analysis

To illustrate how this value could be used in practice, we present an example from a distributional cost‐effectiveness study of two different ways of spending the same fixed budget for increasing uptake of a pre‐existing universal bowel cancer screening programme (Asaria *et al*., 2015). Distributional cost‐effectiveness analysis (DCEA) uses inequality aversion parameters to explore the implications of alternative social value judgements when comparing different policy options that involve trade‐offs between improving total health and reducing health inequality. When using standard cost‐effectiveness analysis, which considers only average quality‐adjusted life year gains, it is more cost‐effective to spend the budget on a ‘Universal’ reminder programme rather than a ‘Targeted’ reminder that reduces health inequality by targeting the most income deprived. As Figure 4 of their paper illustrates, DCEA identifies the threshold level of inequality aversion above which the ‘Targeted’ reminder becomes cost‐effective (*ε* = 8 and *α* = 0.12). Thus, the corresponding parameter values generated in our study (*ε* = 10.95 and *α* = 0.15) suggest that the targeted intervention is the intervention that should be chosen in order to maximise social welfare allowing for inequality aversion. Table 2 illustrates this by computing EDE quality‐adjusted life years per 100 000 for different levels of inequality aversion applied to this example.

**Table 2:** QALY and EDE QALY gains per 100 000 population

	Programme	
	Targeted	Universal
Gains in average QALYs (*ε* = *α* = 0)	3850	4000
Gains in Atkinson EDE QALYs (*ε* = 10.95)	3310	3260
Gains in Kolm EDE QALYs (*α* = 0.15)	3300	3270

## 5 Conclusion

Atkinson's and Kolm's inequality aversion parameters were elicited by using an online survey of members of the general public in England. The vast majority of people, 81.51%, were willing to sacrifice gains in total health in order to reduce health inequality. The responses indicate substantial aversion to health inequality among the English general public, in line with findings from previous studies. If these responses are taken at face value, they imply that marginal health gains to the poorest fifth should be given between 6 and 7 times the weight of health gains to the richest fifth. The inequality aversion parameters elicited provide values that can be used within methods such as DCEA. Through using these methods, societal decision‐makers can evaluate health policies that have the dual objectives of improving population health and reducing health inequality.

## Conflict of Interest

The authors have no conflict of interest.

## Funding Sources

This study was part funded by the C2D2 programme, an inter‐disciplinary initiative supported by the University of York and the Wellcome Trust. Matthew Robson was primarily funded by NIHR and is now supported by the AQM ESRC studentship (ES/J500215/1). Richard Cookson is supported by the NIHR (Senior Research Fellowship, SRF‐2013‐06‐015). The views expressed in this publication are those of the authors and not necessarily those of the funding bodies, the NHS or the Department of Health.

## Ethical Statement

We obtained research ethics approval from the University of York Health Sciences Research Governance Committee, in a letter dated 23 May 2013. The main ethical issues involved informed consent and data security to ensure participant responses were kept anonymous and no personal data disclosed.

## Supporting information

Appendix A: Survey Methods
Appendix B: Response Categorisation
Appendix C: Sample Characteristics
Appendix D: Data Quality and Sensitivity Analysis
Appendix E: Categorised Inequality Aversion Parameters
Appendix F: Restricting Responses due to Duration of Survey

Supporting Information

## References

[hec3430-bib-0001] Abásolo I , Tsuchiya A . 2004 Exploring social welfare functions and violation of monotonicity: an example from inequalities in health ‐ a reply to Jan Abel Olsen. Journal of Health Economics 23: 333–334. 10.1016/j.jhealeco.2003.08.003 15019757

[hec3430-bib-0002] Abásolo I , Tsuchiya A . 2008 Understanding preference for egalitarian policies in health: are age and sex determinants? Applied Economics 40: 2451–2461.

[hec3430-bib-0003] Abásolo I , Tsuchiya A . 2013 Is more health always better for society? Exploring public preferences that violate monotonicity. Theory and Decision 74: 539–563.

[hec3430-bib-0004] Asaria M , Griffin S , Cookson R , Whyte S , Tappenden P . 2015 Distributional cost‐effectiveness analysis of health care programmes ‐ a methodological case study of the UK Bowel Cancer Screening Programme. Health Economics 24: 742–754. 24798212 10.1002/hec.3058

[hec3430-bib-0005] Atkinson AB . 1970 On the measurement of inequality. Journal of Economic Theory 2: 244–263.

[hec3430-bib-0006] Attema AE , Brouwer W , L'haridon O , Pinto‐Prades JL . 2015 Estimating sign‐dependent societal preferences for quality of life. Journal of Health Economics 43: 229–243. 26263893 10.1016/j.jhealeco.2015.07.006

[hec3430-bib-0007] Cookson R , Ali S , Tsuchiya A , Asaria, M . 2015 A040; Value judging, fast and slow: an experimental study of the effects of slow thinking interventions on expressed health inequality aversion. Conference paper presented at the UK *Health Economists*' *Study Group*. Leeds, January 2015.

[hec3430-bib-0008] Dolan P , Tsuchiya A . 2007 Do NHS staff and members of the public share the same views about how to distribute health benefits? Social Science & Medicine 64: 2499–2503. 17449155 10.1016/j.socscimed.2007.03.013

[hec3430-bib-0009] Dolan P , Tsuchiya A . 2009 The social welfare function and individual responsibility: some theoretical issues and empirical evidence from health. Journal of Health Economics 28: 210–220. 19062115 10.1016/j.jhealeco.2008.10.003

[hec3430-bib-0010] Dolan P , Tsuchiya A . 2011 Determining the parameters in a social welfare function using stated preference data: an application to health. Applied Economics 43: 2241–2250.

[hec3430-bib-0011] Edlin R , Tsuchiya A , Dolan P . 2012 Public preferences for responsibility versus public preferences for reducing inequalities. Health Economics 21: 1416–1426. 22072569 10.1002/hec.1799

[hec3430-bib-0012] Essex UO . 2015 Institute for Social and Economic Research and National Centre for Social Research, Understanding Society: Innovation Panel, Waves 1–7, 2008–2014. Colchester, Essex: UK Data Archive.

[hec3430-bib-0014] Kolm SC . 1976 Unequal inequalities.1. Journal of Economic Theory 12: 416–442.

[hec3430-bib-0015] Wagstaff A . 1991 QALYs and the equity‐efficiency trade‐off. Journal of Health Economics 10: 21–41. 10113661 10.1016/0167-6296(91)90015-f

[hec3430-bib-0016] Williams A , Dolan P , Tsuchiya A . 2005 Eliciting equity‐efficiency trade offs in health In *Health Policy and Economics*: *Opportunities and Challenges*, Smith PC, Ginnelly L, Sculpher M (eds.), Open University Press: New York.

