# Supplementary material for: Eliciting the Level of Health Inequality Aversion in England
Source: Health Econ. 2016 Sep 20;26(10):1328–34. doi: 10.1002/hec.3430 (PMC6120144; doi:10.1002/hec.3430)
Supplement: Supplementary file 1 — Supporting Information [file HEC-26-1328-s001.pdf]

APPENDICES

Appendix A: Survey Methods

Survey administration and recruitment were performed by a commercial online survey company, called "SmartSurvey" (https://www.smartsurvey.co.uk). This survey company has a large pre-existing consumer panel of individuals throughout the UK who complete online surveys sponsored by a variety of public, private and academic research organisations. We requested a sample representative of the English general population. Unfortunately, however, the survey company were unable to provide us with response rate information either about selection into the panel from the general public, or about selection into our survey from their panel. The research team designed and created the online questionnaire within web-based software provided by SmartSurvey. Respondents completed the survey from personal computers over the internet. Prior to answering the trade-off questions respondents were shown an e-learning video, designed and piloted by the research team (Cookson et al., 2015). This video was intended to increase understanding of the trade-offs they were to make in a vivid, accessible way. Four animated individuals presented alternative arguments, which represented the equality-efficiency trade-offs that the respondents could make. The video can be accessed at the following link: https://vimeo.com/91930211. After this, each respondent answered additional questions on demographics and social attitudes.

Screenshots of the seven pairwise choices are below; and screenshots of the full online survey are available at: www.york.ac.uk/che/research/equity/economic_evaluation/publicviews/

Figure A1: Online Trade-Off Questions

TRADE OFF QUESTION

Imagine that you are asked to choose between two large government programmes which will improve population health. Both programmes cost exactly the same.

Who Benefits?

Programme	Population Group	Before	Change	After
Programme A	Richest Fifth	74	+7	81
	Poorest Fifth	62	+3	65
Programme B	Richest Fifth	74	+3	77
	Poorest Fifth	62	+8	70

These are gains in years of life in full health over the average person's lifetime.

When making a decision, it is important to remember the following:

- We cannot pay for both programmes - a choice must be made
- "Equally good" means you don't mind which one is chosen
- Both programmes cost exactly the same
- The only difference between the programmes is the gain to the poorest and richest fifth
- The middle three fifths of the population are not affected

Which programme should the government choose?

1.

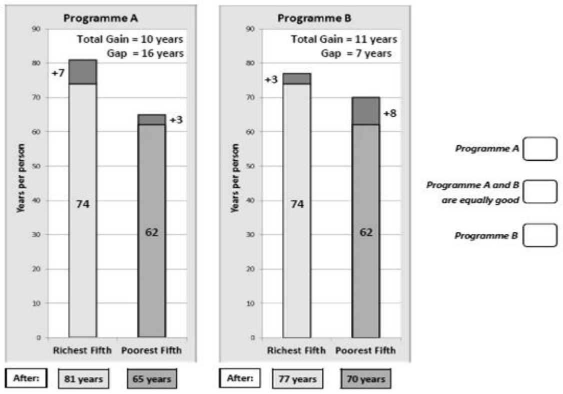

4.

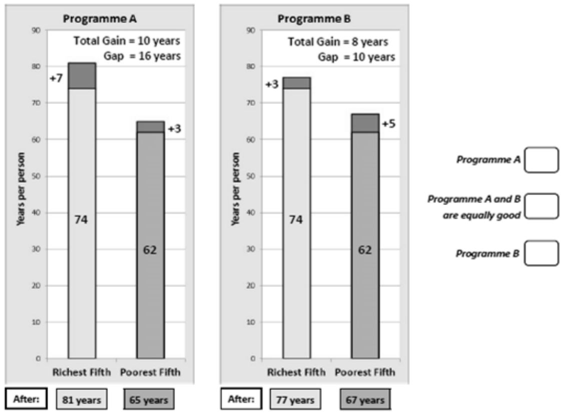

5.

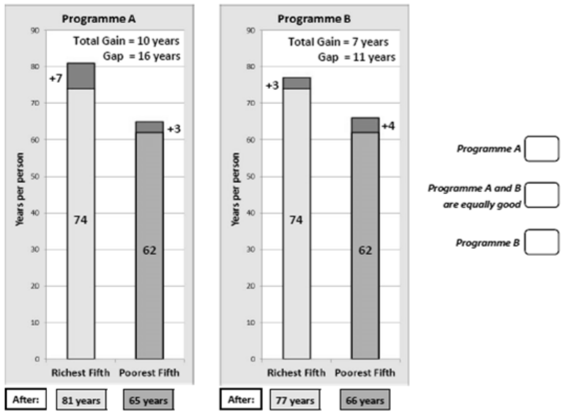

Now imagine it is more difficult than we thought to benefit the poorest fifth.
For each of the following comparisons please tick ONE box per comparison.

2.

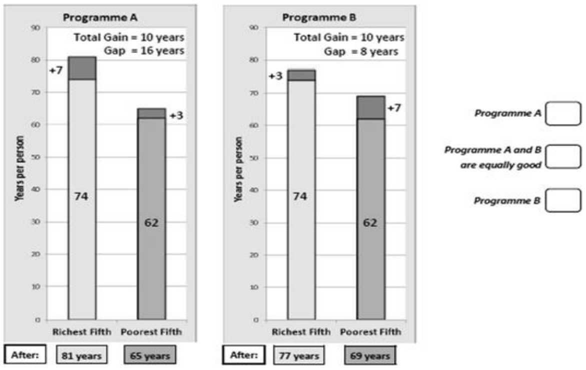

3.

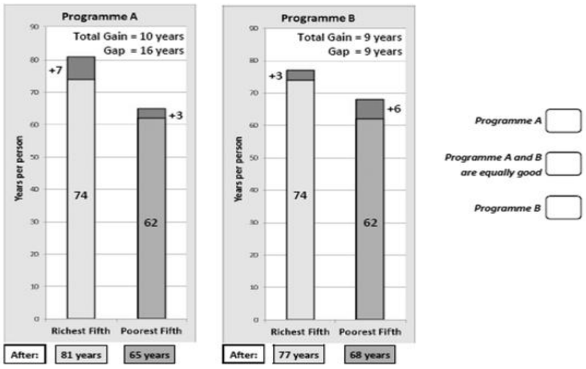

6.

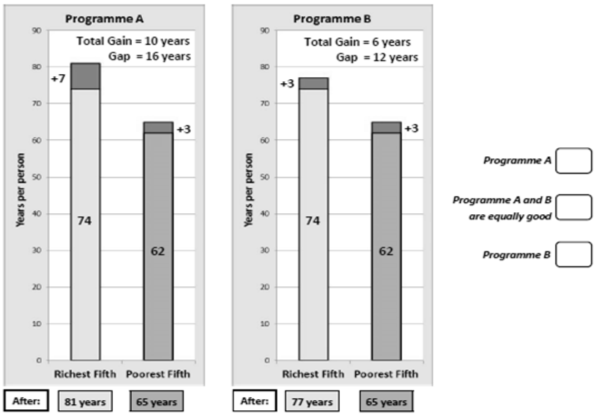

7.

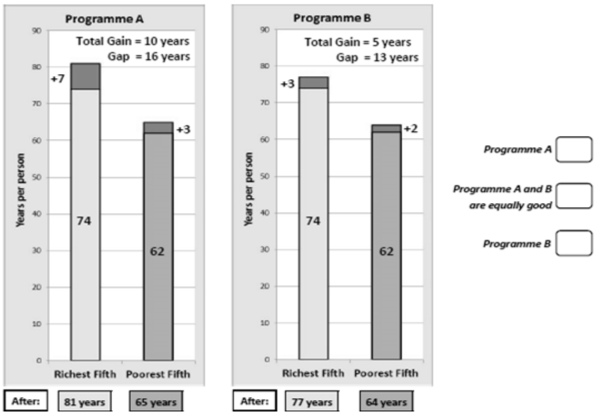

Appendix B: Response Categorisation

Responses are denoted by seven-character sequences representing the seven sequential choices in the questionnaire. A response denoted by "A" shows a preference for Programme A, "B" for Programme B and "=" for an indifference between the two. BBBB=AA, for instance, indicates that the respondent preferred Programme B in the first four choices, was indifferent in the fifth and preferred Programme A in the final two choices.

Table AI: Categorisation of Responses

Rank	Category	Response	Indifferent Between	
			**Health Gains Programme A**	**Health Gains Programme B**
1	Pro-Rich 1	AAAAAAA	7, 3	3, 8.5
2	Pro-Rich 2	=AAAAAA	7, 3	3, 8
3	Pro-Rich 3	BAAAAAA	7, 3	3, 7.5
4	Health Maximiser	B=AAAAA	7, 3	3, 7
5	Weighted Prioritarian 1	BBAAAAA	7, 3	3, 6.5
6	Weighted Prioritarian 2	BB=AAAA	7, 3	3, 6
7	Weighted Prioritarian 3	BBBAAAA	7, 3	3, 5.5
8	Weighted Prioritarian 4	BBB=AAA	7, 3	3, 5
9	Weighted Prioritarian 5	BBBBAAA	7, 3	3, 4.5
10	Weighted Prioritarian 6	BBBB=AA	7, 3	3, 4
11	Weighted Prioritarian 7	BBBBBAA	7, 3	3, 3.5
12	Maximin	BBBBB=A	7, 3	3, 3
13	Egalitarian 1	BBBBBBA	7, 3	3, 2.5
14	Egalitarian 2	BBBBBB=	7, 3	3, 2
15	Egalitarian 3	BBBBBBB	7, 3	3, 1.5

The table shows each of the 15 "logical" responses; where respondents prefer either Programme A or B throughout, or have a single "switching" point. In the cases where respondents "switched" directly between the two programmes, rather than explicitly specifying that they considered the two programmes to be equal, the health gains at the

switching point were assumed to be halfway between the two scenarios on either side of the switch. The point of indifference could not be observed for the Pro-Rich 1 and Egalitarian 3 responses; for these respondents it was assumed that they would have “switched” at the next logical scenario i.e. where the health of the poorest group under Programme B was increased or decreased by another half a year respectively.

Appendix C: Sample Characteristics

This table shows basic descriptive statistics of the sample compared with the English general population, alongside the median response for each particular group. Data for the general population in England are taken from Understanding Society 2014 (Essex., 2015), a large and representative household sample survey.

Table AII: Comparable Sample Compositions; General English Population and Survey Sample (n=244)

	Highest Qualification						Gender	
	Degree	Other Degree	A Level etc	GCSE etc	Other	None	Male	Female
**Pop. in England**	24.60%	11.52%	20.22%	19.96%	10.41%	13.29%	48.29%	51.71%
**Sample**	52.46%	7.79%	13.11%	17.62%	4.92%	4.10%	48.77%	51.23%
**Median Response**	WP 5/6	WP 7	WP 7	WP 7	WP 7	EG 2/3	WP 6	WP 7

	Age Group				Monthly Household Income Before Tax				
	18-34	35-49	50-64	65+	£1000 or Less	£1001 - 1700	£1701 - 2700	£2701 - 4200	£4201 or more
**Pop. in England**	25.69%	26.20%	24.62%	23.84%	7.23%	15.02%	20.73%	24.51%	32.51%
**Sample**	29.10%	22.95%	34.43%	13.52%	28.69%	19.26%	14.34%	13.11%	24.59%
**Median Response**	WP 7	WP 6	WP 7	WP 7	WP 7	WP 7	WP 6	WP 6/7	WP 5

In order to readjust the sample to ensure representativeness weights were generated for each of the four dimensions, resulting in 240 independent weights from our sample and the weighted Understanding Society sample. The Stata command *bsweights* (Kolenikov, 2010) was used to implement a *svy bootstrap*, which executed a bootstrap, for complex survey data. This method allowed the calculation of the 95% confidence intervals for the sample, weighted to reflect a representative sample.

There are differences in median responses by demographic subgroups. The median for those who have obtained at least a degree is slightly less egalitarian in comparison to the median for those who have no degree; while the median for those with no qualifications is within the Egalitarian category. The median for males is found to be less egalitarian than the median for females, while the median across different age groups seem to be stable, with the exception of the median for 35-49 year olds. The median for the most affluent quintile of the sample weights the gains of the worst-off to a lesser degree than the medians of the other four quintiles.

To supplement the observations within Table AI an ordered probit was ran, to test for statistical significance. The categorical rank was the dependant variable, while the demographic characteristics shown, either categorical (Education, Age and Income) or dummy (Gender) variables, were on the right hand side. The results show that differences in both Education and Income are significant to the 5% level, while Age and Gender are insignificant. In both cases, for Education and Income, the lower the level the more egalitarian preferences are.

Appendix D: Data Quality and Sensitivity Analysis

There were 460 respondents who partook in the survey, of whom 216 provided “invalid” data and were excluded from the base case analysis. They consisted of (a) 19 respondents who answered the questions in less than the five minutes it takes to watch the video; (b) 50 who answered “equally good” to every question which shows logical inconsistency of their responses. ; and (c) 147 who provided data that did not fall into our theoretical categorisation. This left 244 for the base case analysis. Two sensitivity analyses were conducted by relaxing exclusion criteria (c).

The first sensitivity analysis included responses that suggest a degree of imprecision; where respondents responded “equally good” in two or more consecutive pairs, with the exception of those within exclusion criteria (b). For responses with two consecutive pairs, the second “equally good” was assumed to be Option B, while for those with more than two consecutive pairs the central “equally good” was assumed to be the switching point. This resulted in including an additional 46 responses. The second sensitivity analysis included responses that suggest a degree of instability, whereby two B-to-A switching points were observed suggesting a possible one-off error that is subsequently corrected (e.g. BBABAAA). In this case, we randomised which switching point to accept as the correct one, which resulted in including a further 8 responses. The median rank did not change in any of these sensitivity analyses, and therefore our value for the inequality aversion parameters, based on this median rank, is robust to these alternative data quality inclusion criteria.

Table AIII: Data Quality Sensitivity Analysis, Sample Sizes and Median Categorical Rank

Sensitivity Analysis	Useable Responses (N = 391)				Median Rank
	"Consistent"		"Inconsistent"		
**Base Case**	**244**	62.40%	**158**	37.60%	11
**First**	**290**	74.17%	**119**	25.83%	11
**Second**	**298**	76.21%	**87**	23.79%	11

Appendix E: Categorised Inequality Aversion Parameters

The specific inequality aversion parameters associated with each response category are given for each of the SWFs in the table below. By inputting the parameters below into each SWF the level of social welfare for specific distributions can be shown for precise levels of inequality aversion.

Table AIV: Parameter Values for each Response Category

Rank	Category	Atkinson's, ε	Atkinson Implied Weight	Kolm's, α	Kolm Implied Weight
1	Pro-Rich 1	-2.075	0.69	-0.028	0.71
2	Pro-Rich 2	-1.419	0.78	-0.019	0.8
3	Pro-Rich 3	-0.731	0.88	-0.01	0.89
4	Health Maximiser	0	1	0	1
5	Weighted Prioritarian 1	0.792	1.15	0.011	1.14
6	Weighted Prioritarian 2	1.671	1.34	0.023	1.32
7	Weighted Prioritarian 3	2.673	1.6	0.037	1.56
8	Weighted Prioritarian 4	3.862	1.98	0.053	1.89
9	Weighted Prioritarian 5	5.358	2.58	0.074	2.43
10	Weighted Prioritarian 6	7.43	3.72	0.103	3.44
11	Weighted Prioritarian 7	10.946	6.95	0.152	6.2
12	Maximin	∞	∞	∞	∞
13	Egalitarian 1	NA	NA	NA	NA
14	Egalitarian 2	NA	NA	NA	NA
15	Egalitarian 3	NA	NA	NA	NA

Appendix F: Restricting Responses due to Duration of Survey

When respondents undertook the survey they were required to watch an e-learning video that lasted 5 minutes and 47 seconds. If participants watched the whole video alongside answering all the questions, the expected time to finish was 15 minutes. Using data on the time taken to complete the survey, respondents who could not have watched the video could be excluded. This provides indirect evidence on whether watching the video had an effect on the responses. Figure A3 shows three distributions of responses: the first is the sample used in the base case analysis (where all respondents took 5 minutes or more); the second where individuals taking less than 10 minutes were further excluded; and the third where those taking less than 15 minutes were further excluded.

Figure A2: Distribution of Responses; Restricting According to Duration

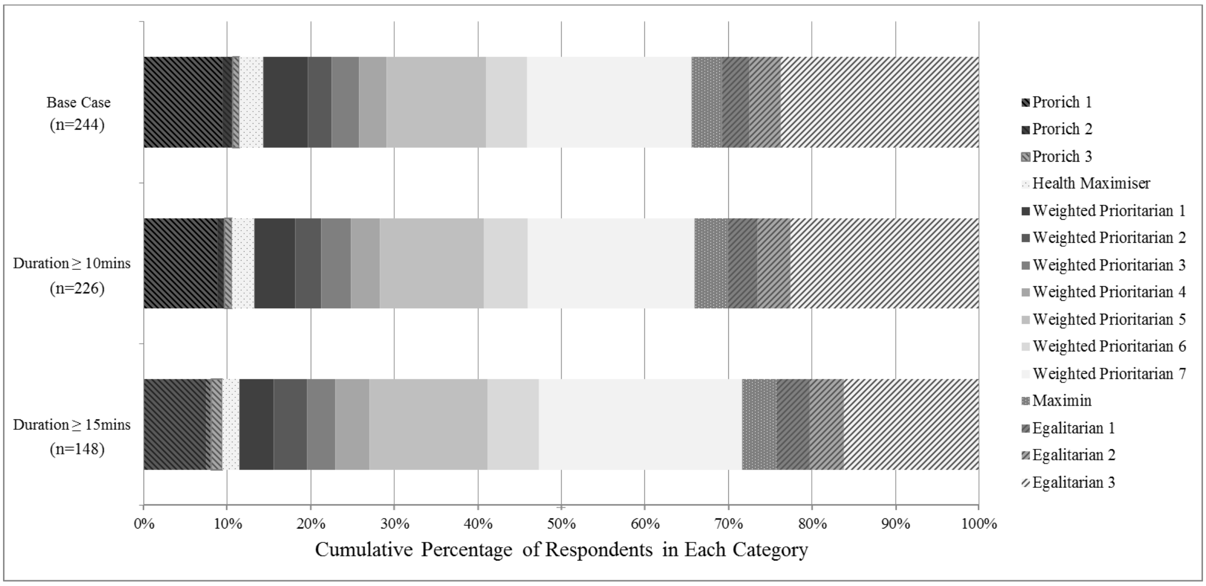

Although the median response remains the same throughout, the greater the exclusion the greater the reduction in ‘extreme’ responses. The Pro-Rich responses reduce from 11.48% to 10.62% to 9.46%, while the Egalitarian responses reduce from 30.74% to 30.09% to 24.32%, as more respondents are excluded. The responses which are not categorised by either Pro-Rich or Egalitarian increase from 57.79% to 59.29% to 66.22%.
